# 

**Published:** 1880-12

**Authors:** 


### DIRECTORY OF DENTAL SOCIETIES.

*American Dental Association*—Meets on the first Tuesday in August, 1880, at Boston, Mass. Pres. L. D. Shephard, Boston, ; Cor. Sec. M. H. Webb, Lancaster, Pa.; Rec. Sec. Geo. A. Cushing, Chicago, Ill.

*American Dental Convention*—Meets in New York, first Tuesday of September, 1880. Pres. Jno. Allen, New York ; Rec. Sec. A. Tees, Philadelphia, Pa.

*National Dental Association*—Meets in New York, on the first Tuesday of September, 1880. Pres. J. B. Patrick, Charleston, S. C.; Cor. Sec. D. E. Evert, Raleigh, North Carolina.

### STATE DENTAL SOCIETIES.

*California State Dental Association*—June 8, 1880. Pres. A. F. McLain, San Rafael Sec. S. E. Goe., San Francisco ; Cor. Sec. H. E. Knox, San Francisco.

*Connecticut State Dental Society*—Pres. E. T. Payne ; Rec. Sec. Geo. L. Parmelee; Hartford. Semi-annual meeting in October, 1880, at Hartford.

*Dental Society of the State of Maryland and District of Columbia*—Meets annually. Next meeting at Washington City, on the second Tuesday in October, 1880. Pres. B. F. Coy, of Baltimore ; Sec. M. W. Foster, M. D.

*Georgia State Dental Society*—Meets second Monday in July, 1880, at Augusta. Pres. W. C. Wardlow, Augusta, Rec. Sec. R. A. Holliday, Atlanta, Cor. Sec. L. D. Carpenter, Atlanta.

*Illinois State Dental Society*—meets annually. Pres. C. A. Kitchen, Rockford ; Sec E. Noyes, Chicago. Next place of meeting, Bloomington, second Tuesday in May, 1880.

*Indiana State Dental Society*—Meets next year at Indianapolis, on the last Tuesday of June, 1880. Pres. W. L. Heiskell, Knightstown; Sec. J. A. Turner, Franklin.

*Iowa State Dental Society*—Meets annually. Next meeting at Oskaloosa, on the first Tuesday after the 4th of July, 1880. Pres. W. P. Dickinson, Dubuque ; Rec. Sec. E. E. Hughes, of Newton ; Cor. Sec. L. C. Ingersoll, Keokuk.

*Kentucky State Dental Association*—Meets annually, first Tuesday in June, 1880. Next meeting in Richmond. Pres. J. S. Cassidy, Covington, Sec. C. G. Edwards, Louisville.

*Kansas State Dental Association*—Pres. A. Doud, Sec. R. J. Pearson, of Atchison: Next meeting will be held at Emporia, first Tuesday in May, 1880.

*Maine Dental Society*—Meets in August, at Thomaston. Pres. E. J. Robert, Agusta, Sec. C. E. Hussey, Beddeford.

*Massachusetts Dental Society*—Meets semi-annually. Pres. J. H. Kidder, of Lawrence; Rec. Sec. Dwight M. Clapp, Boston; Cor. Sec. T. H. Chandler, Boston.

*Michigan State Dental Association*—Meets annually. Next meeting at Detroit, March 31, 1881. Pres. J. C. Parker, Grand Rapids. Sec. M. F. Finley, Ypsilanti. Treas. J. Lathrop, Detroit.

*Mississippi State Dental Association*—Will meet third Wednesday of September, 1880 at Jackson, Pres. A. H. Hilzheim, Jackson ; Sec, M. C. Marshall, Winona.

*Minnesota Dental Association*—Meets annually. Meets May 26, 1880, at Winona. Pres. W. F. Lewis, Sec. C. M. Bailey, Minneapolis.

*Missouri Dental Association*—Meets annually on the first Tuesday of June, 1880. Next meeting at Sweet Springs. Pres. A. N. Fuller; Sec. W. H. Eames, of St. Louis.

*Nebraska State Dental Society*—Meets annually. Next mnW[illegible]. July 23, 1880, at Fremont. Pres. J. W. Chadduck, Nebraska City; Sec.[illegible]etiF. Roseman, Fremont,

*New Jersey State Dental Society*—Meets annually. Next meeting at Long Branch. on Wednesday, July, 1880. Pres. F. A. Levey, Orange; Sec. C. A. Meeker, Newark.

*New Hampshire Dental Society*—Pres. D. W. Eagerly, Farmington; Sec. C. W. Clerment, Manchester.

*North Carolina State Dental Society*—Meets annually. Next meeting at Raleigh, first Wednesday in September, 1880. Pres. V. E. Turner; Sec. D. A. Robinson.

*Northern Ohio Dental Association*—Meets annually. Next meeting at Akron, second Tuesday and Wednesday of May, 1880. Pres J. W. Lyder, Akron; Sec. L. C. Kelsey, Elyria.

*Ohio State Dental Society*—Meets annually at Columbus, first Tuesday of December, 1880. Pres. F. A. Hunter, Cincinnati; Rec. Sec. W. H. Sillito, Xenia; Cor. Sec. E. G. Betty, Cincinnati.

*Oregon State Dental Society*—Meets annually. Pres. J. R. Cardwell, Portland; Rec. Sec. S. J. Barber.

*Pennsylvania State Dental Society*—Meets last Tuesday in July, 1880, at Bellefonte. Pres. W. E. Magill, Erie; Rec. Sec. E. P. Kremer, Lebanon, Pa.; Cor. Sec. G. W. Klump.

*Rhode Island Dental Association*—Pres. A. W. Buckland, Woonsocket; Sec. L. L. Buckland, Providence.

*South Carolina State Dental Association*—Meets annually. Pres. J. B. Patrick, Charleston; Sec. G. F. S. Wright, Columbia; Cor. Sec. B. H. Teagne, Aikin. Next meeting at Charleston, the second Tuesday of July, 1880.

*Tennessee Dental Association*—Meets Semi-annually. Next meeting at Nashville, fourth Wednesday in June, 1880. Pres. A. Bartman; Rec. Sec. L. G. Noel, Nashville; Cor. Sec. W. H. P. Jones.

*Vermont State Dental Society*—Meets annually. Pres. J. L. Perkins, St. Johnsburg; Sec. C. D. Newell, St. Johnsburg.

*Virginia State Dental Association*—Meets in Charlottsville, Tuesday, August 19, 1879 Pres. J. Hal Moore; Sec. L. M. Cowarden, Richmond.

*Wisconsin State Dental Association*—Meets annually. Next meeting at Milwaukee, July 20, 1880. Pres. Wm. Decker, Oshkosh; Sec. M. T. Moore, L a Crosse.

*The Dental Society of the State of New York*—Meets annually on the second Wednesday in May. Next session at Albany, May 12, 1880. Pres. C. E. Francis, New York; Rec. Sec. S. A. Freeman, Buffalo; Cor. Sec. W. H. Atkinson, New York.

### LOCAL SOCIETIES.

*American Academy of Dental Science*—Meets annually in Boston, Mass., on the last Wednesday in October, 1880. Pres. E. G. Tucker; Rec. Sec. E. P. Bradbury; Cor. Sec. E. N. Harris, Boston.

*Brooklyn Dental Society*—Meets monthly. Pres. Chas. D. Cooke; Cor. Sec. W. H. Atkinsyn. Rec. Sec. A. P. Crandall, 508 Clinton Ave., Brooklyn.

*Connecticut Valley Dental Association*—Meets in June, 1880, place undecided. Pres. C. T. Stockwell, Springfield, Mass. Sec. A. M. Ross, Chicopee.

*East Tennessee Dental Association*—Meets annually. Next meeting will be held at Chattanooga, Tenn., on the first Wednesday of Aug., 1880. Pres. S. M. Prothro, Chattanooga; Sec. A. W. Palmer, Kingston.

*Merrimack Valley Dental Association*—Meets annually. Pres. J. N. Chandler, Boston; Sec. H. W. Coburn, Lowell, Mass.

*Mississippi Valley Dental Association*—Meets annually at Cincinnati, on first Wednesday in March, 1881. Pres. N. M. Reid, Cincinnati; Sec. E. G. Betty, Cincinnati.

*Nashville Dental Association*—Meets on the second Tuesday of each month, Pres. R. R. Freeman; Sec. L. G. Noel, Nashville.

*Northern Ohio Dental Association*—Meets annually. Next meeting at Akron, on the second Tuesday in May, 1880. Pres. J. W. Lyder, Akron, O; Cor. Sec. H. F. Harvey, Cleveland.

*Ohio College Dental Association*—Meets annually, Tuesday, March 2, 1880, at Cincinnati. Pres. F. A. Hunter, Cincinnati; Rec. Sec. H. A. Smith, Cincinnati.

*Odontographic Society of Pennsylvania*—Meets on the first Wednesday of each month, at 8 o'clock p. m., in the Philadelphia Dental College. Pres. J. L. Eisenbrey, Rec. Sec. E. L. Hewitt.

*Odontological Society of New York City*—Meets the second Tuesday of each month. Pres. A. L. Northrup; Rec. Sec. Wm. Jarvie, Jr.

*Pennsylvania Association of Dental Surgeons.*—Pres. J. H. Githens. Rec. Sec. Theo. F. Chupein.

*Pittsburg Dental Society*—Meets the second Tuesday Evening of each month. Pres. N. W. Aithen; Sec. L. Depriz.

### EXCHANGES.

St. Louis, Medical and Surgical Journal.
The Pacific Medical and Surgical Journal, San Francisco.
Cincinnati Medical Advance.
Cincinnati Lancet and Clinic.
Dental Cosmos, Philadelphia.
Ladies' Repository, Cincinnati.
Chicago Medical Examiner.
The Detroit Review of Medicine and Pharmacy.
Medical Record, N. Y.
Nashville Journal of Medicine and Surgery.
American Journal of Dental Science, Baltimore.
The United States Medical and Surgical Journal, Chicago.
Canada Journal of Dental Science, Montreal.
Indiana Journal of Medicine.
Missouri Dental Journal, St. Louis.
The Sanitaran, New York City.
The Monthly Review of Dental Surgery, London.
L'art Dentaire, Paris.
Le Progress Dentaire, Paris.
Deutische Verteljahrsschrift, Leipzig.
Physician and Surgeon, Ann Arbor, Mich.

### THE DENTAL COLLEGE
### OF THE
### UNIVERSITY OF MICHIGAN.

The Sixth Annual Session of this Institution will commence on the 1st of October and close on the last Wednesday in March, thus making a course of six months. The regular course of instruction commences at once, and will proceed through the term, with the customary vacation.

The students in this department will receive instructions in Anatomy, Physiology, Pathology, Chemistry, Materia Medica, Therapeutics and Surgery, from the Professors of their respective branches in the *Department of Medicine and Surgery* of the University, when lectures commence, and continue the same as with the Dental College. There will also, in addition, be a special course upon each of these branches. These courses will each embrace from ten to fifteen lectures.

### FACULTY OF THE DENTAL DEPARTMENT.

J. B. ANGELL, LL. D., - - - - - - - President.
J. TAFT, D. D. S., - Principles and Practice of Operative Dentistry.
CORYDON L. FORD, M. D., D. D. S., - - Anatomy and Physiology.
J. A. WATLING, D. D. S., - - Clinical and Mechanical Dentistry.
W. H. DORRANCE, D. D. S., - Demonstrator of Mechanical Dentistry.

### SPECIAL COURSES.

A. B. PALMER, M. D., - - - - - - Dental Pathology.
DONALD MACLEAN, M. D., - - - - - - Oral Surgery.
E. S. DUNSTER, M. D., Diseases of Women and Children with reference to the Teeth.
GEO. E. FROTHINGHAM, M. D., - - - - Dental Therapeutics.

*Geo. L. Field, D D. S, will give Special Instruction in Continuous Gum Work.*

Students should be promptly present at the College on Thursday, September 30th, at 10 o'clock A. M., to make the preliminary arrangements for entering upon regular work on the morning of October 1st. Seats in the lecture room are assigned by selection to students in the order of registration on the Steward's books; and each student is expected to occupy during the session such seat as he may select. Students on arriving at Ann Arbor, should call at the Steward's office.

### CONDITIONS OF GRADUATION.

The candidate must be twenty-one years of age. He must furnish evidence of good moral character.

He must devote three years to the study of his profession. He must attend two full courses of lectures in the Dental College, or one course in some college having an equal standard of requirements, and the last one here, and we recommend that he attend three courses regularly.

He must sustain an examination satisfactory to the Faculty in all the branches taught.

A graduate of the Medical College may enter the senior class, and, if found qualified, may graduate after one year has been devoted to the study of dentistry.

### FEES AND EXPENSES.

The fees, which must be paid in advance, are as follows:

Residents of Michigan.—Matriculation fee, $10.00; annual dues, $20,00.

Non-Residents.—Matriculation, $25.00; annual fees, $25.00.

Graduation Fee.—For all alike, $10.00. The admission fee is paid but once, and entitles the student to the privileges of permanent membership in any department of the University. The annual due is paid the first year and every year thereafter while at the University.

For further particulars, address the Dean of the Dental College, Ann Arbor, Michigan.

May-80, 6 mo. **J. TAFT, Dean.**

### OHIO COLLEGE OF DENTAL SURGERY,

**No. 29 College Street, CINCINNATI, O.**

THIRTY FIFTH ANNUAL SESSION, 1880-81.

### FACULTY.

JAMES TAYLOR, M. D., D. D. S.,
Principles and Practice, and
Dental Hygiene.

WM. CLENDENNIN, M. D.,

Anatomy and Surgery.

J. S. CASSIDY, M. D., D. D. S.,

Chemistry and Materia Medica.

H. A. SMITH, D. D. S.,
Clinical Dentistry.

A. O. RAWLS, D. D. S.,

Pathology and Therapeutics.

J. R. CLAYTON, D. D. S.
Physiology and Histology.

FRANK BELL, D. D. S.,
Mechanical Dentistry and Metallurgy.

G. W. KEELY, D. D. S., Lecturer on Causes & Management of Irregularities of the Teeth
F. H. REHWINKEL, D. D. S., Lecturer on Oral Surgery.
H. L. MOORE, D. D. S., }
J. M. CLYDE, D. D. S., } Demonstrators of Operative Dentistry.
H. E. HIGHLANDS, D. D. S., Demonstrator of Mechanical Dentistry.
OTTO ARNOLD, D. D. S., Demonstrator of Anatomy.
ARTHUR ROSE, D. D. S., Instructor in Physiology.

### CLINICAL INSTRUCTORS.

Dr. F. A. Hunter, Dr. E. Osmond, Dr. J. I. Taylor,
Dr. A. W. Smith, Dr. N. S. Hoff, Dr. F. Peabody,
Dr. D. W. Clancy, Dr. R. G. Richter, Dr. J. K. Jamison,
Dr. E. W. Anderson, Dr. W. S. How, Dr. S. Wardel,
Dr. C. W. Wardle, Dr. C. R. Taft, Dr. C. I. Keely

This Institution is a *Dental College in the strictest sense.* The property is owned by an Association of Dentists, numbering nearly one hundred. The faculty is composed of Practitioners of Dentistry, whose purpose it is to give a thorough course of instruction in the theory and practice of dentistry.

The college building was designed, erected, and is exclusively used for a Dental School. The reception, lecture, clinical and dissecting-rooms, and mechanical laboratory are large, well lighted and admirably arranged for the purpose of dental education.

Located in the center of one of our most popular cities, where there is no other Dental School, the supply of patients is abundant and at times in excess of the requirements for clinical practice.

The College Infirmary will be open every afternoon, (except Sundays,) during the session, thus affording the students ample opportunities to engage in *actual practice* under the direction of the Professors and Demonstrators.

### GRADUATION.

The candidate must be twenty-one years of age. He must have attended two courses of lecturers in a Dental or Medical College, the last of which must be in this institution. A reputable dentist in actual practice five years, may be examined for the degree of D. D. S., after attending one full course of lecturers.

### FEES.

| | |
|---|---|
| Matriculation fee, (but once,) | $5.00 |
| Professors' tickets for one session | 75.00 |
| Dissecting ticket, including material | 10.00 |
| Diploma fee | 20.00 |

The session begins the 12th of October, and continues until the 1st of March. Board can be had for from $4.00 to $6.00 per week.

For further information, address;

**H. A. SMITH, Dean,**

***286 Race Street, CINCINNATI.***

### THE DENTAL COLLEGE
### OF THE
### UNIVERSITY OF MICHIGAN.

The Sixth Annual Session of this Institution will commence on the 1st of October and close on the last Wednesday in March, thus making a course of six months. The regular course of instruction commences at once, and will proceed through the term, with the customary vacation.

The students in this department will receive instructions in Anatomy, Physiology, Pathology, Chemistry, Materia Medica, Therapeutics and Surgery, from the Professors of their respective branches in the *Department of Medicine and Surgery* of the University, when lectures commence, and continue the same as with the Dental College. There will also, in addition, be a special course upon each of these branches. These courses will each embrace from ten to fifteen lectures.

### FACULTY OF THE DENTAL DEPARTMENT.

J. B. ANGELL, LL. D., - - - - - - - President.
J. TAFT, D. D. S., - Principles and Practice of Operative Dentistry.
CORYDON L. FORD, M. D., D. D. S., - - Anatomy and Physiology.
J. A. WATLING, D. D. S., - - Clinical and Mechanical Dentistry.
W. H. DORRANCE, D. D. S., - Demonstrator of Mechanical Dentistry.

### SPECIAL COURSES.

A. B. PALMER, M. D., - - - - - - Dental Pathology.
DONALD MACLEAN, M. D., - - - - - - Oral Surgery.
E. S. DUNSTER, M. D., Diseases of Women and Children with reference to the Teeth.
GEO. E. FROTHINGHAM, M. D., - - - - Dental Therapeutics.

*Geo. L. Field, D. D. S., will give Special Instruction in Continuous Gum Work.*

Students should be promptly present at the College on Thursday, September 30th, at 10 o'clock A. M., to make the preliminary arrangements for entering upon regular work on the morning of October 1st. Seats in the lecture room are assigned by selection to students in the order of registration on the Steward's books; and each student is expected to occupy during the session such seat as he may select. Students on arriving at Ann Arbor, should call at the Steward's office.

### CONDITIONS OF GRADUATION.

The candidate must be twenty-one years of age. He must furnish evidence of good moral character.

He must devote three years to the study of his profession. He must attend two full courses of lectures in the Dental College, or one course in some college having an equal standard of requirements, and the last one here, and we recommend that he attend three courses regularly.

He must sustain an examination satisfactory to the Faculty in all the branches taught.

A graduate of the Medical College may enter the senior class, and, if found qualified, may graduate after one year has been devoted to the study of dentistry.

### FEES AND EXPENSES.

The fees, which must be paid in advance, are as follows:

Residents of Michigan.—Matriculation fee, $10.00; annual dues, $20,00.

Non-Residents.—Matriculation, $25.00; annual fees, $25.00.

Graduation Fee.—For all alike, $10.00. The admission fee is paid but once, and entitles the student to the privileges of permanent membership in any department of the University. The annual due is paid the first year and every year thereafter while at the University.

For further particulars, address the Dean of the Dental College, Ann Arbor, Michigan.

6 mo. **J. TAFT, Dean.**

### OHIO COLLEGE OF DENTAL SURGERY,

**No. 29 College Street, CINCINNATI, O.**

THIRTY FIFTH ANNUAL SESSION, 1880-81.

### FACULTY.

JAMES TAYLOR, M. D., D. D. S.,
Principles and Practice, and
Dental Hygiene.

WM. CLENDENNIN, M. D.,

Anatomy and Surgery.

J. S. CASSIDY, M. D., D. D. S.,

Chemistry and Materia Medica.

H. A. SMITH, D. D. S.,
Clinical Dentistry.

A. O. RAWLS, D. D. S.,

Pathology and Therapeutics.

J. R. CLAYTON, D. D. S.
Physiology and Histology.

FRANK BELL, D. D. S.,
Mechanical Dentistry and Metallurgy.

G. W. KEELY, D. D. S., Lecturer on Causes & Management of Irregularities of the Teeth.
F. H. REHWINKEL, D. D. S., Lecturer on Oral Surgery.
H. L. MOORE, D. D. S., } Demonstrators of Operative Dentistry.
J. M. CLYDE, D. D. S., }
H. E. HIGHLANDS, D. D. S., Demonstrator of Mechanical Dentistry.
OTTO ARNOLD, D. D. S., Demonstrator of Anatomy.
ARTHUR ROSE, D. D. S., Instructor in Physiology.

### CLINICAL INSTRUCTORS.

Dr. F. A. Hunter, Dr. E. Osmond, Dr. J. I. Taylor,
Dr. A. W. Smith, Dr. N. S. Hoff, Dr. F. Peabody,
Dr. D. W. Clancy, Dr. R. G. Richter, Dr. J. K. Jamison,
Dr. E. W. Anderson, Dr. W. S. How, Dr. S. Wardel,
Dr. C. W. Wardle, Dr. C. R. Taft, Dr. C. I. Keely

This Institution is a *Dental College in the strictest sense.* The property is owned by an Association of Dentists, numbering nearly one hundred. The faculty is composed of Practitioners of Dentistry, whose purpose it is to give a thorough course of instruction in the theory and practice of dentistry.

The college building was designed, erected, and is exclusively used for a Dental School. The reception, lecture, clinical and dissecting-rooms, and mechanical laboratory are large, well lighted and admirably arranged for the purpose of dental education.

Located in the center of one of our most popular cities, where there is no other Dental School, the supply of patients is abundant and at times in excess of the requirements for clinical practice.

The College Infirmary will be open every afternoon, (except Sundays,) during the session, thus affording the students ample opportunities to engage in *actual practice* under the direction of the Professors and Demonstrators.

### GRADUATION.

The candidate must be twenty-one years of age. He must have attended two courses of lecturers in a Dental or Medical College, the last of which must be in this institution. A reputable dentist in actual practice five years, may be examined for the degree of D. D. S., after attending one full course of lecturers.

### FEES.

Matriculation fee, (but once,) .......... $5.00
Professors' tickets for one session .......... 75.00
Dissecting ticket, including material .......... 10.00
Diploma fee .......... 20.00

The session begins the 12th of October, and continues until the 1st of March. Board can be had for from $4.00 to $6.00 per week.

For further information, address

**H. A. SMITH, Dean,**

***286 Race Street, CINCINNATI.***

### THE DENTAL COLLEGE

### OF THE

### UNIVERSITY OF MICHIGAN.

The Sixth Annual Session of this Institution will commence on the 1st of October and close on the last Wednesday in March, thus making a course of six months. The regular course of instruction commences at once, and will proceed through the term, with the customary vacation.

The students in this department will receive instructions in Anatomy, Physiology, Pathology, Chemistry, Materia Medica, Therapeutics and Surgery, from the Professors of their respective branches in the *Department of Medicine and Surgery* of the University, when lectures commence, and continue the same as with the Dental College. There will also, in addition, be a special course upon each of these branches. These courses will each embrace from ten to fifteen lectures.

### FACULTY OF THE DENTAL DEPARTMENT.

J. B. ANGELL, LL. D., - - - - - - - - President.
J. TAFT D. D. S., - Principles and Practice of Operative Dentistry.
CORYDON L. FORD, M. D., D. D. S., - - Anatomy and Physiology.
J. A. WATLING, D. D. S., - - Clinical and Mechanical Dentistry.
W. H. DORRANCE, D. D. S., - Demonstrator of Mechanical Dentistry.

### SPECIAL COURSES.

A. B. PALMER, M. D., - - - - - - Dental Pathology.
DONALD MACLEAN, M. D., - - - - - - Oral Surgery.
E. S. DUNSTER, M. D., Diseases of Women and Children with reference to the Teeth.
GEO. E. FROTHINGHAM, M. D., - - - - - Dental Therapeutics.

*Geo L. Field, D. D. S., will give Special Instruction in Continuous Gum Work.*

Students should be promptly present at the College on Thursday, September 30th, at 10 o'clock A. M., to make the preliminary arrangements for entering upon regular work on the morning of October 1st. Seats in the lecture room are assigned by selection to students in the order of registration on the Steward's books; and each student is expected to occupy during the session such seat as he may select. Students on arriving at Ann Arbor, should call at the Steward's office.

### CONDITIONS OF GRADUATION.

The candidate must be twenty-one years of age. He must furnish evidence of good moral character.

He must devote three years to the study of his profession. He must attend two full courses of lectures in the Dental College, or one course in some college having an equal standard of requirements, and the last one here, and we recommend that he attend three courses regularly.

He must sustain an examination satisfactory to the Faculty in all the branches taught.

A graduate of the Medical College may enter the senior class, and, if found qualified, may graduate after one year has been devoted to the study of dentistry.

### FEES AND EXPENSES.

The fees, which must be paid in advance, are as follows:

RESIDENTS OF MICHIGAN.—Matriculation fee, $10.00; annual dues, $20,00.

NON-RESIDENTS.—Matriculation, $25.00; annual fees, $25.00.

GRADUATION FEE.—For all alike, $10.00. The admission fee is paid but once, and entitles the student to the privileges of permanent membership in any department of the University. The annual due is paid the first year and every year thereafter while at the University.

For further particulars, address the Dean of the Dental College Ann Arbor, Michigan.

May 80 6 mo. **J. TAFT, Dean.**

### OHIO COLLEGE OF DENTAL SURGERY,

**No. 29 College Street, CINCINNATI, O.**

THIRTY FIFTH ANNUAL SESSION, 1880-81.

### FACULTY.

JAMES TAYLOR, M. D., D. D. S.,
Principles and Practice, and
Dental Hygiene.

WM. CLENDENNIN, M. D.,

Anatomy and Surgery.

J. S. CASSIDY, M. D., D. D. S.,

Chemistry and Materia Medica.

H. A. SMITH, D. D. S.,
Clinical Dentistry.

A. O. RAWLS, D. D. S.,

Pathology and Therapeutics.

J. R. CLAYTON, D. D. S.
Physiology and Histology.

FRANK BELL, D. D. S.,
Mechanical Dentistry and Metallurgy.

G. W. KEELY, D. D. S., Lecturer on Causes & Management of Irregularities of the Teeth.
F. H. REHWINKEL, D. D. S., Lecturer on Oral Surgery.
H. L. MOORE, D. D. S., } Demonstrators of Operative Dentistry.
J. M. CLYDE, D. D. S., }
H. E. HIGHLANDS, D. D. S., Demonstrator of Mechanical Dentistry.
OTTO ARNOLD, D. D. S., Demonstrator of Anatomy.
ARTHUR ROSE, D. D. S., Instructor in Physiology.

### CLINICAL INSTRUCTORS.

Dr. F. A. Hunter, Dr. E. Osmond, Dr. J. I. Taylor,
Dr. A. W. Smith, Dr. N. S. Hoff, Dr. F. Peabody,
Dr. D. W. Clancy, Dr. R. G. Richter, Dr. J. K. Jamison,
Dr. E. W. Anderson, Dr. W. S. How, Dr. S. Wardel,
Dr. C. W. Wardle, Dr. C. R. Taft, Dr. C. I. Keely

This Institution is a *Dental College in the strictest sense.* The property is owned by an Association of Dentists, numbering nearly one hundred. The faculty is composed of Practitioners of Dentistry, whose purpose it is to give a thorough course of instruction in the theory and practice of dentistry.

The college building was designed, erected, and is exclusively used for a Dental School. The reception, lecture, clinical and dissecting-rooms, and mechanical laboratory are large, well lighted and admirably arranged for the purpose of dental education.

Located in the center of one of our most popular cities, where there is no other Dental School, the supply of patients is abundant and at times in excess of the requirements for clinical practice.

The College Infirmary will be open every afternoon, (except Sundays,) during the session, thus affording the students ample opportunities to engage in *actual practice* under the direction of the Professors and Demonstrators.

### GRADUATION.

The candidate must be twenty-one years of age. He must have attended two courses of lecturers in a Dental or Medical College, the last of which must be in this institution. A reputable dentist in actual practice five years, may be examined for the degree of D. D. S., after attending one full course of lecturers.

### FEES.

| | |
|---|---|
| Matriculation fee, (but once,) | $5.00 |
| Professors' tickets for one session | 75.00 |
| Dissecting ticket, including material | 10.00 |
| Diploma fee | 20.00 |

The session begins the 12th of October, and continues until the 1st of March. Board can be had for from $4.00 to $6.00 per week.

For further information, address

**H. A. SMITH, Dean,**

***286 Race Street, CINCINNATI.***

### THE DENTAL COLLEGE
### OF THE
### UNIVERSITY OF MICHIGAN.

The Sixth Annual Session of this Institution will commence on the 1st of October and close on the last Wednesday in March, thus making a course of six months. The regular course of instruction commences at once, and will proceed through the term, with the customary vacation.

The students in this department will receive instructions in Anatomy, Physiology, Pathology, Chemistry, Materia Medica, Therapeutics and Surgery, from the Professors of their respective branches in the *Department of Medicine and Surgery* of the University, when lectures commence, and continue the same as with the Dental College. There will also, in addition, be a special course upon each of these branches. These courses will each embrace from ten to fifteen lectures.

### FACULTY OF THE DENTAL DEPARTMENT.

J. B. ANGELL, LL. D., - - - - - - - - President.
J. TAFT, D. D. S., - Principles and Practice of Operative Dentistry.
CORYDON L. FORD, M. D., D. D. S., - - Anatomy and Physiology.
J. A. WATLING, D. D. S., - - Clinical and Mechanical Dentistry.
W. H. DORRANCE, D. D. S., - Demonstrator of Mechanical Dentistry.

### SPECIAL COURSES.

A. B. PALMER, M. D., - - - - - - - Dental Pathology.
DONALD MACLEAN, M. D., - - - - - - - Oral Surgery.
E. S. DUNSTER, M. D., Diseases of Women and Children with reference to the Teeth.
GEO. E. FROTHINGHAM, M. D., - - - - - Dental Therapeutics.

*Geo L. Field, D. D. S., will give Special Instruction in Continuous Gum Work.*

Students should be promptly present at the College on Thursday, September 30th, at 10 o'clock A. M., to make the preliminary arrangements for entering upon regular work on the morning of October 1st. Seats in the lecture room are assigned by selection to students in the order of registration on the Steward's books; and each student is expected to occupy during the session such seat as he may select. Students on arriving at Ann Arbor, should call at the Steward's office.

### CONDITIONS OF GRADUATION.

The candidate must be twenty-one years of age. He must furnish evidence of good moral character.

He must devote three years to the study of his profession. He must attend two full courses of lectures in the Dental College, or one course in some college having an equal standard of requirements, and the last one here, and we recommend that he attend three courses regularly.

He must sustain an examination satisfactory to the Faculty in all the branches taught.

A graduate of the Medical College may enter the senior class, and, if found qualified, may graduate after one year has been devoted to the study of dentistry.

### FEES AND EXPENSES.

The fees, which must be paid in advance, are as follows:

RESIDENTS OF MICHIGAN.—Matriculation fee, $10.00; annual dues, $20,00.

NON-RESIDENTS.—Matriculation, $25.00; annual fees, $25.00.

GRADUATION FEE.—For all alike, $10.00. The admission fee is paid but once, and entitles the student to the privileges of permanent membership in any department of the University. The annual due is paid the first year and every year thereafter while at the University.

For further particulars, address the Dean of the Dental College, Ann Arbor, Michigan.

May 80 6 mo. **J. TAFT, Dean.**

### OHIO COLLEGE OF DENTAL SURGERY,

**No. 29 College Street, CINCINNATI, O.**

THIRTY FIFTH ANNUAL SESSION, 1880-81.

### FACULTY.

JAMES TAYLOR, M. D., D. D. S.,
Principles and Practice, and
Dental Hygiene.

WM. CLENDENNIN, M. D.,

Anatomy and Surgery.

J. S. CASSIDY, M. D., D. D. S.,

Chemistry and Materia Medica.

H. A. SMITH, D. D. S.,
Clinical Dentistry.

A. O. RAWLS, D. D. S.,

Pathology and Therapeutics.

J. R. CLAYTON, D. D. S.
Physiology and Histology.

FRANK BELL, D. D. S.,
Mechanical Dentistry and Metallurgy.

G. W. KEELY, D. D. S., Lecturer on Causes & Management of Irregularities of the Teeth.
F. H. REHWINKEL, D. D. S., Lecturer on Oral Surgery.
H. L. MOORE, D. D. S., J. M. CLYDE, D. D. S., Demonstrators of Operative Dentistry.
H. E. HIGHLANDS, D. D. S., Demonstrator of Mechanical Dentistry.
OTTO ARNOLD, D. D. S., Demonstrator of Anatomy.
ARTHUR ROSE, D. D. S., Instructor in Physiology.

### CLINICAL INSTRUCTORS.

Dr. F. A. Hunter, Dr. E. Osmond, Dr. J. I. Taylor,
Dr. A. W. Smith, Dr. N. S. Hoff, Dr. F. Peabody,
Dr. D. W. Clancy, Dr. R. G. Richter, Dr. J. K. Jamison,
Dr. E. W. Anderson, Dr. W. S. How, Dr. S. Wardel,
Dr. C. W. Wardle, Dr. C. R. Taft, Dr. C. I. Keely

This Institution is a *Dental College in the strictest sense.* The property is owned by an Association of Dentists, numbering nearly one hundred. The faculty is composed of Practitioners of Dentistry, whose purpose it is to give a thorough course of instruction in the theory and practice of dentistry.

The college building was designed, erected, and is exclusively used for a Dental School. The reception, lecture, clinical and dissecting-rooms, and mechanical laboratory are large, well lighted and admirably arranged for the purpose of dental education.

Located in the center of one of our most popular cities, where there is no other Dental School, the supply of patients is abundant and at times in excess of the requirements for clinical practice.

The College Infirmary will be open every afternoon, (except Sundays,) during the session, thus affording the students ample opportunities to engage in *actual practice* under the direction of the Professors and Demonstrators.

### GRADUATION.

The candidate must be twenty-one years of age. He must have attended two courses of lecturers in a Dental or Medical College, the last of which must be in this institution. A reputable dentist in actual practice five years, may be examined for the degree of D. D. S., after attending one full course of lecturers.

### FEES.

| | |
|---|---|
| Matriculation fee, (but once,) | $5.00 |
| Professors' tickets for one session | 75.00 |
| Dissecting ticket, including material | 10.00 |
| Diploma fee | 20.00 |

The session begins the 12th of October, and continues until the 1st of March. Board can be had for from $4.00 to $6.00 per week.

For further information, address

**H. A. SMITH, Dean,**

***286 Race Street, CINCINNATI.***

### THE DENTAL COLLEGE

OF THE

### UNIVERSITY OF MICHIGAN.

The Sixth Annual Session of this Institution will commence on the 1st of October and close on the last Wednesday in March, thus making a course of six months. The regular course of instruction commences at once, and will proceed through the term, with the customary vacation.

The students in this department will receive instructions in Anatomy, Physiology, Pathology, Chemistry, Materia Medica, Therapeutics and Surgery, from the Professors of their respective branches in the *Department of Medicine and Surgery* of the University, when lectures commence, and continue the same as with the Dental College. There will also, in addition, be a special course upon each of these branches. These courses will each embrace from ten to fifteen lectures.

### FACULTY OF THE DENTAL DEPARTMENT.

J. B. ANGELL, LL. D., - - - - - - - President.
J. TAFT D. D. S., - Principles and Practice of Operative Dentistry.
CORYDON L. FORD, M. D., D. D. S., - - Anatomy and Physiology.
J. A. WATLING, D. D. S., - - Clinical and Mechanical Dentistry.
W. H. DORRANCE, D. D. S., - Demonstrator of Mechanical Dentistry.

### SPECIAL COURSES.

A. B. PALMER, M. D., - - - - - - Dental Pathology.
DONALD MACLEAN, M. D., - - - - - - Oral Surgery.
E. S. DUNSTER, M. D., Diseases of Women and Children with reference to the Teeth.
GEO. E. FROTHINGHAM, M. D., - - - - Dental Therapeutics.

*Geo L. Field, D. D. S., will give Special Instruction in Continuous Gum Work.*

Students should be promptly present at the College on Thursday, September 30th, at 10 o'clock A. M., to make the preliminary arrangements for entering upon regular work on the morning of October 1st. Seats in the lecture room are assigned by selection to students in the order of registration on the Steward's books; and each student is expected to occupy during the session such seat as he may select. Students on arriving at Ann Arbor, should call at the Steward's office.

### CONDITIONS OF GRADUATION.

The candidate must be twenty-one years of age. He must furnish evidence of good moral character.

He must devote three years to the study of his profession. He must attend two full courses of lectures in the Dental College, or one course in some college having an equal standard of requirements, and the last one here, and we recommend that he attend three courses regularly.

He must sustain an examination satisfactory to the Faculty in all the branches taught.

A graduate of the Medical College may enter the senior class, and, if found qualified, may graduate after one year has been devoted to the study of dentistry.

### FEES AND EXPENSES.

The fees, which must be paid in advance, are as follows:

RESIDENTS OF MICHIGAN.—Matriculation fee, $10.00; annual dues, $20,00.

NON-RESIDENTS.—Matriculation, $25.00; annual fees, $25.00.

GRADUATION FEE.—For all alike, $10.00. The admission fee is paid but once, and entitles the student to the privileges of permanent membership in any department of the University. The annual due is paid the first year and every year thereafter while at the University.

For further particulars, address the Dean of the Dental College Ann Arbor, Michigan.

May 80 6 mo. **J. TAFT, Dean.**

### CONTENTS.

### COMMUNICATIONS.

A MONTHLY MAGAZINE DEVOTED TO THE INTERESTS OF

**THE DENTAL PROFESSION.**

*TERMS REDUCED TO $2.50 PER YEAR. SINGLE COPIES 25C.*

EDITED BY PROF. J. TAFT, D.D.S.

AND

*Published by SPENCER, CROCKER & CO., Ohio Dental Depot.*

The volume commences in January and closes in December.

The REGISTER being issued monthly is always fresh, therefore Indispensable to the practitioner who desires to keep posted up with the new inventions and improvements which are daily developed. Neither expense nor effort will be spared to give value and variety to its contents. Articles will be illustrated with well executed engravings whenever they will add to the interest or assist to explanation.

Its extensive circulation and frequency of issue make it one of the BEST DENTAL ADVERTISING MEDIUMS in the United States.

All subscriptions must begin with January or July.

Local or special notices, five lines or less, will be inserted for $3 each insertion; over five lines, 50 cts. per line.

All advertising bills payable in advance, or at furthest, after the issue of the first number containing the advertisement. All transient advertisements to be paid for in advance.

☞CONTRIBUTIONS SOLICITED.

Exclusively Editorial Communications should be addressed to Editor.

The latest number of the REGISTER may be ordered with or without other goods at any time, and all letters should be addressed to

*SPENCER, CROCKER & CO., Ohio Dental Depot, Cincinnati, O.*

